# Plastid genome and its phylogenetic implications of Asiatic *Spiraea* (Rosaceae)

**DOI:** 10.1186/s12870-023-04697-8

**Published:** 2024-01-03

**Authors:** Shu-Yan Zhang, Hai-Fei Yan, Lei Wei, Tong-Jian Liu, Lin Chen, Gang Hao, Xing Wu, Qiao-Ling Zhang

**Affiliations:** 1 College of Life Sciences, South China Agricultural University, Guangzhou, 510642 China; 2 Key Laboratory of Plant Resources Conservation and Sustainable Utilization, South China Botanical Garden, Chinese Academy of Sciences, Guangzhou, 510650 China; 3 South China National Botanical Garden, Guangzhou, 510650 China; 4 Guangdong Provincial Key Laboratory of Applied Botany, South China Botanical Garden, Chinese Academy of Sciences, Guangzhou, 510650 China; 5 Hangzhou Xixi National Wetland Park Service Center (Hangzhou Xixi National Wetland Park Ecology & Culture Research Center), Hangzhou, 310013 China

**Keywords:** *Spiraea*, Phylogeny, Time divergence, Chloroplast genome, Positive selection

## Abstract

**Background:**

*Spiraea* L. is a genus comprising approximately 90 species that are distributed throughout the northern temperate regions. China is recognized as the center of species diversity for this genus, hosting more than 70 species, including 47 endemic species. While *Spiraea* is well-known for its ornamental value, its taxonomic and phylogenetic studies have been insufficient.

**Results:**

In this study, we conducted sequencing and assembly of the plastid genomes (plastomes) of 34 Asiatic *Spiraea* accessions (representing 27 Asiatic *Spiraea* species) from China and neighboring regions. The *Spiraea* plastid genome exhibits typical quadripartite structures and encodes 113–114 genes, including 78–79 protein-coding genes (PCGs), 30 tRNA genes, and 4 rRNA genes. Linear regression analysis revealed a significant correlation between genome size and the length of the SC region. By the sliding windows method, we identified several hypervariable hotspots within the *Spiraea* plastome, all of which were localized in the SC regions. Our phylogenomic analysis successfully established a robust phylogenetic framework for *Spiraea*, but it did not support the current defined section boundaries. Additionally, we discovered that the genus underwent diversification after the Early Oligocene (~ 30 Ma), followed by a rapid speciation process during the Pliocene and Pleistocene periods.

**Conclusions:**

The plastomes of *Spiraea* provided us invaluable insights into its phylogenetic relationships and evolutionary history. In conjunction with plastome data, further investigations utilizing other genomes, such as the nuclear genome, are urgently needed to enhance our understanding of the evolutionary history of this genus.

**Supplementary Information:**

The online version contains supplementary material available at 10.1186/s12870-023-04697-8.

## Background

*Spiraea* L., a member of the tribe Spiraeeae of Amygdaloideae (Rosaceae), comprises approximately 90 species (http://www.plantsoftheworldonline.org), and is distributed across the northern temperate regions of the world. China is recognized as the center of diversity for this genus, with over 70 species, including 47 endemic species [1]. *Spiraea* is renowned for its ornamental value, particularly its attractive white, pink or purple flowers that bloom profusely in spring or summer, forming heads composed of numerous tiny blooms. Additionally, certain varieties of *Spiraea* with golden foliage, such as *Spiraea* × *vanhouttei* ‘Pink Ice’ and ‘Gold Fountain’, have been developed. To fully exploit their horticultural value, a comprehensive taxonomic and phylogenetic study of *Spiraea* taxa is necessary. However, the phylogeny of *Spiraea* has not been extensively investigated thus far.

The traditional infrageneric classification of *Spiraea* is primarily based on inflorescence types, including panicles, compound corymbs, corymb, umbel, and fascicle, as established by Poyarkova [2], Rehder [3], and Yü and Kuan [4]. For Chinese *Spiraea* taxa, four sections (i.e., sects. *Spiraea*, *Calospira*, *Chamaedryon*, and *Glomerati*) and ten series within the genus have been widely accepted [4]. Previous phylogenetic analyses have revealed issues with traditional classifications based on inflorescence morphology, as the majority of sections are considered paraphyletic or polyphyletic [5–10]. However, most nodes in these phylogenetic trees could not receive strong support values based on limited molecular fragments, highlighting the need for the development of robust molecular markers within *Spiraea*.

The plastid, an essential organelle in plant cells, plays a crucial role in plant growth and development [11]. Compared to nuclear and mitochondrial genomes, the plastid genome possesses unique characteristics, such as uniparental inheritance, absence of recombination, conserved genome structure and gene content, and moderate evolutionary rate. These features make the plastid genome highly valuable for resolving phylogenetic relationships at various taxonomic levels [12]. Furthermore, the assembly of plastid genomes is relatively straightforward due to their high cellular copy numbers and the development of assembly algorithms [13]. As a result, the number of complete plastid genomes has significantly increased in recent years, thanks to the advancement of high-throughput DNA sequencing technologies. Plastid genomes have proven to be invaluable in elucidating the phylogenetic backbone of Rosaceae and uncovering its evolutionary history [14]. The number of plastid genomes within the Rosaceae family has also rapidly increased in recent years (e.g., *Fragaria* [15, 16], *Rubus* [17], and *Rosa* [18, 19]), providing a stronger understanding of phylogenetic relationships at the species level and the phylogeographical patterns within the family. However, a limited number of plastid genomes of *Spiraea* species have been reported [20–23], and no solid divergence times of the genus have been inferred. Therefore, it is crucial to conduct comparative analyses with more *Spiraea* plastid genomes to better identify the evolutionary pattern of the plastid genome within *Spiraea* and assess its phylogenetic relationships.

In this study, we assembled the complete plastomes for 34 *Spiraea* accessions representing 27 Asiatic species using several available *Spiraea* plastomes from GenBank. Our objectives were to (1) examine the variation in plastome structure and features of *Spiraea*, (2) identify the hypervariable loci across these *Spiraea* plastomes, (3) infer the phylogenomic relationships of *Spiraea*, and (4) determine the divergence time of *Spiraea*. This study will contribute to a better understanding of the evolution of *Spiraea* based on plastome sequences.

## Materials and methods

### Plant materials, DNA extraction, and sequencing

In this study, we collected a total of 27 Asiatic *Spiraea* species and 34 accessions, representing four sections (i.e., sects. *Calospira*, *Chamaedryon*, *Glomerati* and *Spiraea*) of two subgenera (i.e., subgs. *Protospiraea* and *Metaspiraea*) in *Spiraea.* Among these accessions, 25 were newly collected from Hangzhou Botanical Garden. Voucher specimens were formally identified by Qiao-Ling Zhang and deposited at the Herbarium of South China Botanical Garden (IBSC), Chinese Academy of Sciences (CAS). Silica-gel dried leaves were used for DNA extraction by the cetyltrimethylammonium bromide (CTAB) method [24]. Paired-end (PE) sequencing libraries were constructed and sequenced on an MGISEQ-2000 (MGI, Shenzhen, China) at the Beijing Genomics Institution (BGI, Wuhan, China). Additionally, twelve plastid genomes of *Spiraea* were directly downloaded from GenBank (Accessed on February 5, 2023), which belong to nine *Spiraea* species and three outgroups (i.e., *Malus baccata* (L.) Borkh., *Rosa cymose* Tratt. and *Sibiraea angustata* (Rehd.) Hand.-Mazz.) for phylogenetic inferences. Among all accessions, four *Spiraea* species (*Spiraea japonica* L.F., *S.* × *vanhouttei* (Birot) Zabel, *S. cantoniensis* Lour*.*, and *S. prunifolia* Siebold & Zucc.) have multiple individuals. The detailed sampling information is summarized in Table S1.

### Plastid genome assembly, annotation, and comparison

Plastomes of 25 *Spiraea* accessions were newly assembled using GetOrangelle [25] with the reference plastome of *Spiraea mongolica* (NC_051992). These complete plastid sequences were annotated using the Plastid Genome Annotator (PGA) [26] and manually checked and adjusted with the aid of Geneious 2019 [27]. The circular physical maps were drawn using OrganellarGenome DRAW (ORDRAW) [28].

The total length of the plastomes, as well as the lengths of the large single copy (LSC), small single copy (SSC), and inverted repeat regions (IR_A_ and IR_B_), gene content, and GC content of all *Spiraea* species were analyzed using Geneious. The relationships among the genome size and LSC length, IR length, intron length, and intergenic spacer length of *Spiraea* species were tested using least squares linear regression analysis in R 4.2.2 [29]. The boundary information between the IRs and SC regions was analyzed in Geneious.

### Divergent hotspot analyses

The plastid genome sequences were aligned using MAFFT v7.308 [30]. The nucleotide polymorphism (*Pi*) of plastid genomes was calculated using the sliding window method with a length of 600 bp and a step size of 200 bp in DnaSP v6 [31]. The sequence heterogeneity within 34 *Spiraea* plastid genomes was visualized using mVISTA with the Shuffle-LAGAN model [32].

### Phylogenetic analyses and molecular dating

Phylogenetic analysis was performed on all 34 *Spiraea* plastid genome sequences, with *M. baccata* (MK571561), *R. cymosa* (NC_051550), and *Si*. *Angustata* (NC_054238) used as outgroups. The 79 protein-coding genes (PCGs) and whole plastid genome sequences (including all coding and non-coding regions) were aligned separately using MAFFT and concatenated into a sequence matrix using AMAS [33]. Maximum likelihood (ML) and Bayesian inference (BI) trees were each inferred using RAxML v8.2.10 [34] and MrBayes v3.2.6 [35]. The ML tree was constructed using RAxML under the GTRGAMMA model with 1,000 bootstrap replicates. BI was performed with MrBayes in the GTR + G + I model on XSEDE (the CIPRES Science Gateway, [36]). The Markov chain Monte Carlo (MCMC) chains were run for 10 × 10^6^ generations, and stationarity was assessed using TRACER v1.7.1 (effective sample size (ESS) > 200) [37]. The first 25% of runs were discarded as burn-in, and the remaining trees were used to construct the consensus tree. Bayesian posterior probabilities (BPPs) were used to estimate support for each branch. The phylogenetic tree file was visualized in FigTree v1.4.3 [38].

To estimate the divergence time of *Spiraea*, we retrieved the other 86 plastid genomes of Rosales taxa from GenBank (Table S1). A total of 78 PCGs extracted from 120 plastid genomes were extracted and aligned separately by MAFFT. Maximum likelihood trees were constructed for each of these 78 PCGs using RAxML, and molecular clock-like genes (clock-like genes) were screened from the 78 gene trees by using the SortaDate package [39]. The top-ten clock-like genes were selected and aligned using MAFFT, and then concatenated using AMAS for further divergence time estimation.

The concatenated matrix was used to estimate the divergence time using BEAST v2.7 [40]. The yule tree prior was used with an optimized relaxed clock (ORC) and GTR + GAMMA was selected for the molecular clock model. We ran four independent analyses, each consisting of 400 million generations and sampled every 10,000 generations. The results from these analyses were combined using the LogCombiner package. Convergence was assessed using TRACER, based on the effective sample size (ESS) of each parameter with a likelihood > 200, following the method described in [41]). After discarding the first 25% of trees as burn-in, the maximum clade credibility (MCC) tree was generated using the TreeAnnotator package. The mean nodal heights and age estimation with 95% highest posterior densities (HPDs) were visualized using FigTree.

Twelve reliable fossils from Rosaceae were used for calibration, as described by Xiang et al. [42]. The fossil information and calibration settings are shown in Table S2. Notably, *Spiraea-*like fossils (fruits and leaves) have been found in the Early Eocene upland floras of the Okanogan Highlands of northeastern Washington State and British Columbia, Canada [42, 43]. Therefore, the stem age of *Spiraea* was estimated using a standard lognormal prior distribution with a mean of 0.25, a deviation of 0.3, and an offset of 48 Ma, which roughly matched the hypothesized age (49–50 Ma).

To examine the general patterns of diversification through time of *Spiraea*, lineage-through-time (LTT) plots were calculated using APE v3.5 [44]. To account for uncertainty in the dating estimates, the 1,000 random trees from the 4,000 converged trees from BEAST were used to calculate the 95% confidence interval (CI), and all outgroups were removed.

### Selection pressure analyses

We excluded PCGs with lengths less than 300 bp. A total of 50 PCGs were chosen and aligned using the MUSCLE algorithm [45]. Stop codons were removed from the aligned sequences using MEGA 11 [46]. The resulting aligned sequences were then converted to PML format using PhyloSuite v1.2.2 [47].

To calculate the non-synonymous substitution rate (*d*_N_), synonymous substitution rate (*d*_S_), and ratio values (ω, *d*_N_/*d*_S_) of each protein-coding gene, we employed the Site Model (M0) using EasycodeML v1.4 [48]. The PCGs were categorized into seven functional groups based on the classification by Wicke et al. [49]: ATP synthase, NADPH dehydrogenase, cytochrome b/f complex, photosystem, ribosomal proteins, RNA polymerase, and other genes.

Likelihood ratio tests (LRT) were conducted to determine the presence/absence of selection. The log-likelihood statistic was calculated as 2∆*L* = 2(*L*_1_- *L*_0_). This value was then compared with a χ^2^ distribution with degrees of freedom calculated from the difference in the number of parameters between the models. *L*_0_ represents the log-likelihood under the null model, while *L*_1_ represents the likelihood under the alternative model. Model M0 assumes a single value of *ω* for all sites and lineages, while model M3 includes a discrete distribution of *ω* values. Model M1 assumes two site classes, and was compared with M2, which allows for three site classes including positive selection. Models M7 and M8 were also compared, with M7 assuming a beta distribution for variation in *ω* and M8 allowing for positive selection. Positive selection can only be inferred when models M2, M3, or M8 indicate codons with a *ω* ratio > 1 and the likelihood ratio test for positive selection is significant at *P* < 0.05. Bayesian empirical Bayes (BEB) was used to identify sites potentially under positive selection.

## Results

### Structural features and genome composition of plastid genomes in *Spiraea*

**Fig. 1:**
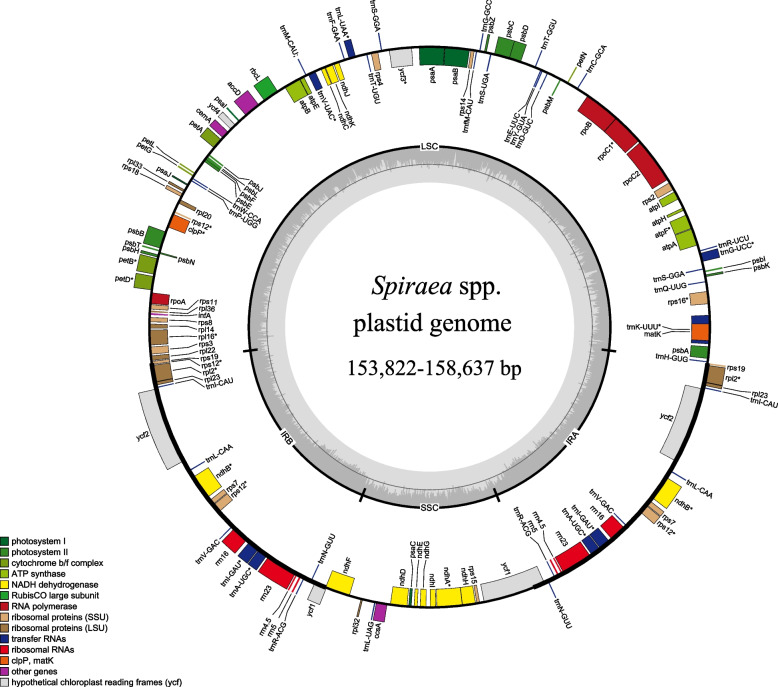
The plastid genome map of Asiatic *Spiraea* species. The genes belonging to different functional groups are shown in different colors. Genes inside and outside of the external circle are transcribed in clockwise and counterclockwise directions, respectively. The inner circle represents the quadripartite structure, with two copies of the inverted repeat (IR_A_ and IR_B_), an LSC, and an SSC region in black with GC content in dark gray and AT content in light gray

**Fig. 2:**
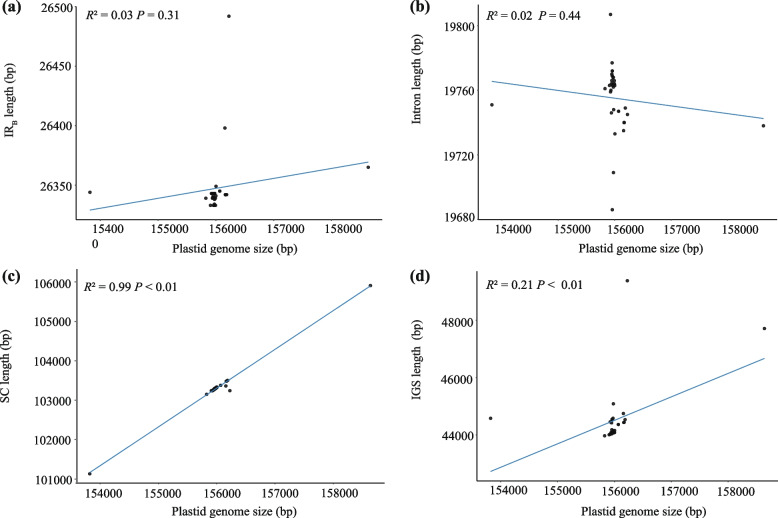
The relationships between the length of different parts of plastome and plastome size within *Spiraea*. **a** Correlation of the IR length and plastome size. **b** Correlation of the intron length and plastome size. **c** Correlation of the SC length and plastome size. **d** Correlation of the IGS length and plastome size

In this study, we analyzed the plastid genomes of 34 individuals from the *Spiraea* genus, representing 27 different Asiatic species. We obtained nine *Spiraea* plastid genome sequences from GenBank. The complete plastid genomes exhibited the typical quadripartite circular structure, consisting of a large single-copy (LSC) region, a small single-copy (SSC) region, and two inverted repeat (IR) regions (Fig. 1). The length of the genomes ranged from 153,822 bp (*S. japonica*, MZ981784) to 158,637 bp (*S. insularis*), while the LSC region length ranged from 82,226 bp (*S. japonica*) to 86,997 bp (*S. insularis*). The shortest SSC region was 26,333 bp, found in four species: *S. ovalis*, *S. henryi*, *S. nipponica*, and *S. trichocarpa*, while the longest SSC region was 26,492 bp in *S. martini*. The IR region length ranged from 18,700 bp (*S. laeta*) to 18,957 bp (*S. ovalis*). The GC content of the *Spiraea* plastid genome ranged from 36.6% to 36.9%. This information is summarized in Table S3. Linear regression analysis showed a significant correlation between genome size and the length of the SC region (*R*^2^ = 0.99, *P* < 0.01) and intergenic spacer (IGS) length (*R*^2^ = 0.21, *P* < 0.01), but no correlation with the IR region (*R*^2^ = 0.03, *P* = 0.31) or intron length (*R*^2^ = 0.02, *P* = 0.44) (Fig. 2).

The *Spiraea* plastid genomes encoded a total of 113–114 genes, including 78–79 protein-coding genes (PCGs), 30 tRNA genes, and 4 rRNA genes (Table S3). Seventeen genes contained one intron, namely, *atpF*, *clpP*, *petB*, *petD*, *ndhA*, *ndhB*, *rpoC1*, *rpl2*, *rpl16*, *rps12*, *rps16*, *trnA-UGC*, *trnI-GAU*, *trnG-UCC*, *trnK-UUU*, *trnL-UAA*, and *trnV-UAC*. One gene, *ycf3*, contained two introns. Additionally, seventeen genes were located in the IR region, including *ndhB*, *rpl2*, *rpl23*, *rps7*, *rps12*, *rrn4.5*, *rrn5*, *rrn16*, *rrn23*, *trnA-UGC*, *trnI-CAU*, *trnI-GAU*, *trnL-CAA*, *trnN-GUU*, *trnR-ACG*, *trnV-GAC*, and *ycf2* (Table S4). The *psaA* gene was absent in the accession of *S. japonica* var. *japonica* (MZ981784).

### Plastid genome variations within *Spiraea*

**Fig. 3:**
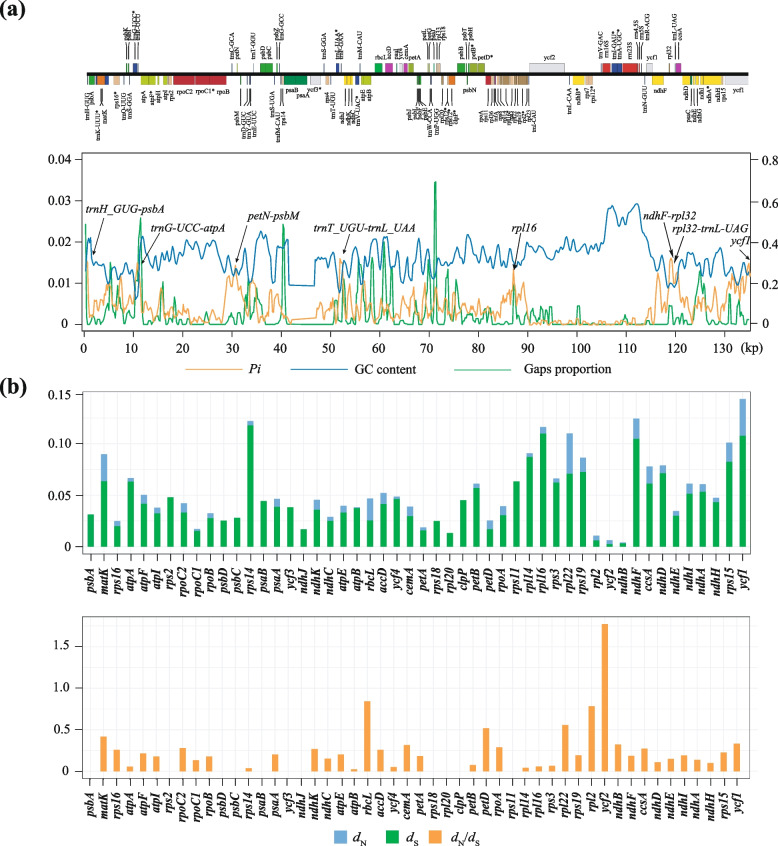
Basic features of *Spireae* plastid genomes. **a** The nucleotide diversity (*Pi*), GC content, and gap proportion in the plastid genome sequences of *Spiraea* detected by sliding window analysis, using 600 bp windows and a 200 bp step size. **b** Nonsynonymous (*d*_N_) and synonymous (*d*_S_) substitution rates and *d*_N_/*d*_S_ for each protein-coding gene within the *Spiraea* plastid genomes

Our study revealed a significant level of conservation in the genome structure, gene order, and gene content of all *Spiraea* plastid genomes. Figure S1 demonstrates that the inverted repeat (IR) region exhibited relatively stable characteristics compared to the large single copy (LSC) and small single copy (SSC) regions, with strikingly higher GC content and lower nucleotide and gap variations (Fig. 3a). The conservation of all rRNA genes was observed, along with certain protein-coding genes, such as *psbA*, *atpA*, *rpoB*, *psbC*, *psbD*, *rps4*, *rbcL*, *accD*, *petA*, *psbB*, *ycf2*, *ndhD*, and *rps7*.

In the comprehensive analysis of 34 *Spiraea* species’ plastid genomes, we observed that the length of the *rps19* gene ranged from 279 to 282 bp, all of which were located in the LSC-IR_B_ region (Fig. S2). Among them, a segment of 108 bp to 252 bp extended into the IR_B_ region, resulting in the duplication of the *rps19* gene in the IR_A_ region. The *ycf1* gene was situated in the SSC-IR_A_ region, with a length ranging from 1,046 bp to 1,064 bp in the IR_A_ region. However, the total length of the *ycf1* gene in *S. martini* was only 1,131 bp, with only 82 bp in the SSC region, while *S. veitchii* exhibited the longest length of 4,577 bp. Additionally, a duplication of the *ycf1* pseudogene was observed in the IR_B_ region. The position of the *ndhF* gene varied, with 22 *Spiraea* accessions harboring it in the SSC region, while it appeared in the IR_B_-SSC region in the remaining accessions. The conserved *trnH-GUG* gene, spanning 74 bp to 75 bp, was distributed in the SSC-IR_A_ region (Fig. S2).

Sliding window analysis demonstrated that nucleotide polymorphisms in *Spiraea* plastid genomes were more conserved in the IR region than in the SC region, and non-coding regions exhibited more pronounced variation than coding regions (Fig. 3a). The nucleotide polymorphism (*Pi*) ranged from 0 to 0.0138, with an average value of 0.0036. Relatively high *Pi* values (> 0.011) were observed in *ndhF-rpl32*, *trnH-GUG-psbA*, *trnG-UCC-atpA*, *petN-psbM*, *trnT-UGU-trnL-UAA*, *rpl16*, *rpl32-trnL-UAG*, and *ycf1*.

### Selection pressure analyses

This study investigated the adaptive evolution of *Spiraea* by analyzing the ratio of *d*_N_/*d*_S_. A ratio greater than one indicates positive selection, a ratio less than one indicates purifying selection, and a ratio equal to one indicates neutral evolution. Analysis of 50 PCGs revealed that the average value of *d*_N_ was 0.0074, with the highest value observed for *rpl22* (0.0394) and the lowest being 0. The average value of *d*_S_ was 0.0449, with the highest value observed for *rps14* (0.1179) and the lowest values observed for *ycf2* (0.0022). The average value of *d*_N_/*d*_S_ was 0.2122, with the highest value observed for *ycf2* (1.7749) and the lowest being 0.0001 (Fig. 3b).

**Fig. 4:**
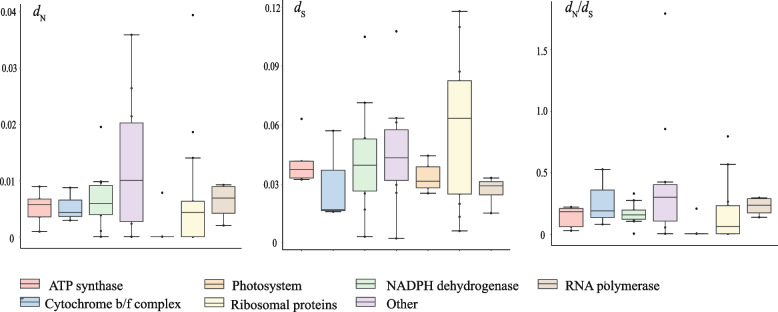
Boxplot of the values of the nonsynonymous (*d*_N_) and synonymous (*d*_S_) substitution rates and *d*_N_/*d*_S_ in each functional gene group

Most genes exhibited a significantly larger *d*_S_ compared to *d*_N_, indicating purifying selection. However, the *d*_N_/*d*_S_ ratio of the *ycf2* gene (1.7749) was greater than one, suggesting that this gene has undergone significant positive selection (*P* < 0.05; Tables S5-6). When categorizing the protein-coding genes based on their function, it was found that the other type genes had the highest *d*_N_ value (0.0127), while photosystem genes had the lowest *d*_N_ value (0.0016). In terms of *d*_S_, ribosomal protein genes had the highest value (0.0599), while RNA polymerase genes had the lowest value (0.0265). Except for the *ycf2* gene whose *d*_N_/*d*_S_ value was greater than 1, the rest of the functional genes exhibited values less than 1. Among these groups, other type genes had the highest *d*_N_/*d*_S_ value (0.4261), while photosystem genes had the lowest (0.0401) (Fig. 4).

### Phylogenetic analyses and divergence time estimates

**Fig. 5:**
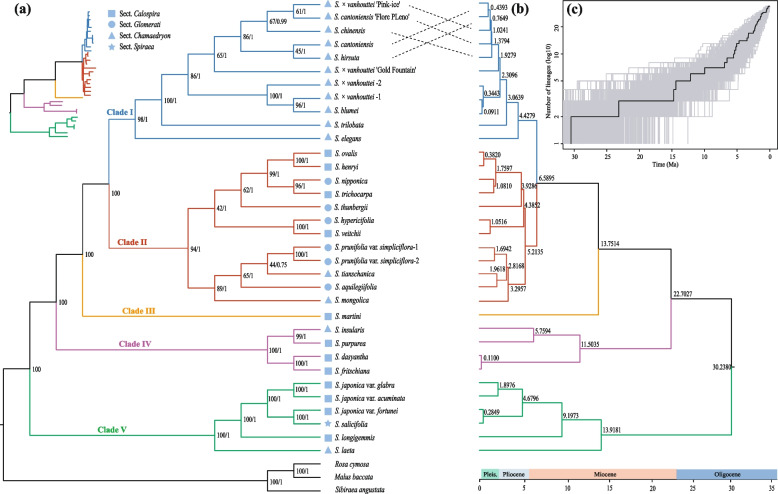
Phylogeny, divergence time estimate, and LTT plot for *Spiraea*. **a** Cladogram of the maximum likelihood (ML) phylogenetic tree based on 79 protein-coding genes (PCGs). All main clades in both trees (ML and BI) are identical. Node labels are marked with the bootstrap values and Bayesian posterior probabilities (bootstrap value/posterior probability). The upper left shows the ML tree with branch length. **b** Maximum clade-credibility tree (MCC tree) from BEAST. Node labels represent estimated divergence times in millions of years. The confidence intervals are provided in Fig. S4. **c** Lineage-through-time plot for *Spiraea*

For this study, a total of 34 *Spiraea* accessions and three outgroups were selected to construct a maximum likelihood (ML) tree and Bayesian inference using the protein-coding genes (PCGs) and whole plastid genome sequences. The ML tree, Bayesian inference, and BEAST MCC tree based on PCGs showed similar phylogenetic topographies, as shown in Fig. 5. Five main clades were identified with high support in the ML tree (Fig. 5a). However, the whole plastid genome sequences only recovered four of these main clades with full support values (Fig. S3). Clade II, identified by PCGs, could not be recovered, and its three branches within the clade were not strongly supported (Fig. S3). Overall, the traditionally defined subgenera and sections of *Spiraea* could not be supported by any datasets or phylogenetic methods (Fig. 5a-b; Fig. S3; Table S1).

To estimate the divergence date, the top-ten clock-like genes were filtered out using Sortadate. These genes were *accD, atpA, matK, ndhA, ndhD, ndhF, ccsA, rpoB, rpoC2*, and *ycf1*. Twelve reliable fossils from Rosaceae were used. The stem age of *Spiraea* was estimated to be 49.24 Ma (95% HPD: 48.64–50.09 Ma), with diversification occurring since the Early Oligocene (~ 30 Ma, Fig. S4). Most extant *Spiraea* lineages (29 out of 34) originated from the Pliocene period, and 25 taxa diverged from their most recent common ancestor (MRCA) since the Pleistocene (Fig. 5b). The LTT plot showed a clear shift in lineages since approximately 5 Ma, followed by a rapid accumulation after approximately 2 Ma (Fig. 5c).

## Discussion

### Plastid genome features and molecular variation patterns of *Spiraea*

The plastid is a vital organelle in plant cells, playing a crucial role in plant growth and development [50]. While significant changes in plastome organization have been observed in various distantly related plant groups, such as conifers, the IR-lacking clade (IRLC) of Fabaceae, and some parasitic flowering plants [51], the plastid genome tends to remain relatively stable across major plant lineages. It typically consists of a quadripartite structure, with two copies of a large inverted repeat (IR) separated by small (SSC) and large (LSC) single-copy regions [51]. The typical plastome organization is also found in *Spiraea* (Fig. 1; Table S1). In our study, we examined the plastid genome of *Spiraea* and identified several variation patterns within these genomes.

First, we observed a positive relationship between the size of SC region and plastid genome size. While expansion/contraction of the IR regions is one major driver of genome size variation [51], this was not evident in *Spiraea* (*R*^2^ = 0.03, *P* = 0.31; Fig. 2a). However, there was a significant correlation between the plastid genome size and the length of the SC region (*R*^2^ = 0.99, *P* < 0.01; Fig. 2c). The gene content across all *Spiraea* plastid genomes was highly conserved, except for *Spiraea japonica* var. *japonica* (accession no. MZ981784), which had the smallest plastid genome within *Spiraea* and lacked an important photosynthesis-related gene, *psaA*. Additionally, the intergenic spacer (IGS) in the SC region may contribute to the size variation of the SC region in *Spiraea* (Fig. 2d).

Second, we identified several hypervariable hotspots within the *Spiraea* plastome, all of which were localized in the SC regions (Fig. 3a). The pattern of lower IR substitution rates compared to the SC regions has been observed in *Spiraea* plastid genomes (Fig. 3a; Fig. S1), as well as in other plant lineages [51]. This decreased substitution rate in the IR is likely a result of a copy-dependent repair mechanism [52, 53].

Previous studies have used nrDNA ITS sequence data and two plastid DNA loci (*trnL-trnF* and *matK*) to investigate the phylogeny of *Spiraea* [6–9]. However, these methods have shown limited resolution in resolving the phylogeny of *Spiraea* [6–9], indicating low sequence divergence. Our results partially support this hypothesis, as *trnL-trnF* and *matK* exhibit relatively low sequence divergence (each with *Pi* values of 0.0043 and 0.0083) compared to highly variable hotspots (such as hotspots with *Pi* values > 0.011) (Fig. 3a). Therefore, hypervariable loci are needed to increase the phylogenetic resolution in low-level phylogenetic or phylogeographic studies in *Spiraea*.

We conducted an investigation into the hypervariable hotspots across the plastid genomes within the *Spiraea* genus. These findings provide valuable genetic resource information for future phylogenetic and phylogeographic studies. We identified eight top hypervariable loci with *Pi* values larger than 0.011, namely, *ndhF-rpl32*, *trnH*-*GUG*-*psbA*, *trnG-UCC-atpA*, *petN*-*psbM*, *trnT*-*UGU*-*trnL*-*UAA*, *rpl16*, *rpl32-trnL-UAG*, and *ycf1*.

Many of these loci, such as *ndhF-rpl32*, *trnH*-*GUG*-*psbA, trnT*-*UGU*-*trnL*-*UAA rpl16*, *rpl32ni-trnL-UAG,* and *ycf1*, have been previously identified in studies conducted by Taberlet et al. [54], Shaw et al. [55–57], and Dong et al. [58]. However, it is important to consider the characteristics of these variable regions themselves. Factors such as fragment size and mutation rate (substitution saturation) can have a detrimental effect on phylogenetic analysis. For example, the *trnH-psbA* locus is controversial due to its high mutation rate and significant length variation caused by insertions, deletions, simple sequence repeats, and localized inversions in some plant lineages [59–61]. These factors can impact the accuracy of sequence alignment and subsequently affect the precision of phylogenetic inference and species identification. Within *Spiraea*, the *trnH-psbA* alignment matrix has 89 indels, indicating its unstable size. Additionally, the *ycf1* locus also exhibits a high degree of sequence variability and has been proposed as a candidate barcode for land plants. However, its high mutation rate and absence in certain plant lineages, such as Poaceae [58, 62], can lead to alignment ambiguities similar to those observed in *trnH-psbA* [58, 63].

Third, we investigated the pattern of molecular evolution among plastid genes in *Spiraea*. The evolution of substitution rates among plastid genes has been extensively studied with the recent release of numerous plastid genomes. Generally, non-synonymous rates of photosynthesis-related genes differ significantly from those of housekeeping genes, indicating that photosynthesis-related genes are under stronger functional constraints [64–66]. Our results demonstrated that the photosynthesis-related genes in terms of *d*_N_ are highly conserved, providing further support for this hypothesis (Fig. 4).

It is generally assumed that most plant species have low substitution rates of most genes in organelles, and these genes evolve under strong purifying selection. However, certain genes in the plastid have undergone positive selection, particularly those involved in photosynthesis and other metabolic pathways (e.g., [67]). The relationship between the high levels of non-synonymous rate evolution and potential selective factors, such as novel ecological conditions, is still a topic of debate [68]. In our study, we observed *d*_N_/*d*_S_ ratios of 1.7749 for the *ycf2* gene, which is likely experiencing strong positive selection (*P* < 0.05; Table S6). Despite its unclear function, *ycf2* is known as the largest chloroplast gene in angiosperms and encodes a motor protein that generates ATP for inner membrane translocation [69] and plant cell survival [70]. Positive selection of the *ycf2* gene has been observed in numerous species (e.g., [71–74]), indicating its adaptive importance in various environments and plant lineages.

### Phylogenetic and evolutionary implications of plastid genomes within *Spiraea*

The inheritance of plastid genomes in plants is typically uniparental, with the mother being the primary contributor. This results in a smaller effective population size compared to nuclear genomes. Consequently, the coalescent time of plastid haplotypes is shorter, making them ideal for studying genetic variations and tracing plastid evolutionary history [75]. Plastid markers, such as *rbcL*, *matK*, and *trnL-trnF*, have been widely used in phylogenetic studies at different taxonomic levels, including ordinal, familial, tribal, and generic levels [12, 76]. They have also been used in lower-level studies, such as phylogeographic investigations [77, 78] and species identification [79, 80].

Previous studies on the phylogeny of *Spiraea* have utilized nrDNA ITS sequence data and limited plastid DNA loci (*trnL-trnF* and *matK*) [6–9]. However, the resolution of the phylogenetic relationships within *Spiraea* based on ITS and *trnL-trnF* is limited, and none of the sections defined by Rehder [3] and Yü and Lu [81] based on inflorescence morphology are monophyletic [8]. Yu et al. [9] largely supported this hypothesis, except for sect. *Spiraea*. The inclusion of *Spiraea douglasii* in sect. *Spiraea* is questionable and represents a clear difference between the two aforementioned studies [8, 9]. The *Spiraea* species sampled in our study represent all four sections found in China (Table S1; [1, 81]). Our phylogenomic analysis provides the most detailed phylogenetic backbone of *Spiraea* to date and strongly supports the notion that the traditionally defined sections of *Spiraea* are not monophyletic groups [8, 9], despite only including one species (*S*. *salicifolia*) from sect. *Spiraea* in this study. We also found a complex evolutionary history of *Spiraea*, as there are many shallow relationships within Clades I and II that could not be highly supported (Fig. 5; Fig. S3). Additionally, some taxa with multiple accessions (such as *S. japonica*,* S. cantoniensis*, and *S*. × *vanhouttei*) are recovered as paraphyletic or polyphyletic groups (Fig. 5; Fig. S3).

There may be two primary reasons why the traditional taxonomic classification and some species boundaries within *Spiraea* cannot be supported by plastid data. First, the phylogenetic relationships inferred from plastid data could not fully capture the evolutionary relationships among taxa, potentially due to the occurrence of pervasive hybridization/introgression events in *Spiraea*. Recent evidence suggests that ancient hybridization/introgression events may have resulted in cytonuclear conflicts [82]. *Spiraea* is a genus known to frequently undergo hybridization, with numerous hybrids arising through horticultural practices [83]. Although wild hybrids are less commonly reported, there have been instances of hybrids occurring across different sections of the genus [83, 84]. These findings indicate that reproductive isolation is not complete among *Spiraea* species, making hybridization a potentially common phenomenon. Consequently, a substantial amount of nuclear genetic data is necessary to further investigate the traditional classification framework and hybridization/introgression events.

Second, more detailed revisions of the infrageneric classification and species boundary delimitation of *Spiraea* are needed. It is evident that the traditional classification system within *Spiraea* is inconsistent with the results obtained from plastid data ([23]; this study), nuclear ribosomal DNA internal transcribed spacer (nrDNA ITS) sequences [6, 7], and their combined analyses [8, 9]. This inconsistency strongly suggests a problem with the traditional infrageneric classification of *Spiraea*. Traditionally, inflorescence morphology has been the main focus in these classification schemes [3, 81], but it is likely that this trait does not accurately reflect the true evolutionary relationships within *Spiraea*. Therefore, an integrative framework for infrageneric classification in the genus is needed, which should incorporate additional diagnostic traits such as seed and palynology evidence [9].

Ancient *Spiraea* taxa are believed to have originated in the Early Eocene based on macrofossil evidence [42, 43]. Our study is the first to identify the divergence sequence of the Asian *Spiraea* taxa using twelve reliable fossil calibrations of Rosaceae. We acknowledge that the crown age inferred in this study may have been underestimated due to our limited sampling. Nevertheless, our divergence time for the genus is much older than the result of *Spiraea* taxa in the Qinghai-Tibetan Plateau (QTP) [6], which can be attributed to the difference in molecular markers, divergence estimate methods, and the species included in the two studies ([6]; this study). We conclude that *Spiraea* diversified after the Early Oligocene (~ 30 Ma), followed by a rapid speciation process during the Pliocene and Pleistocene periods (Fig. 5b-c). The Pliocene–Pleistocene period played a vital role in the speciation process of *Spiraea*, as most extant taxa used in this study originated during this period (Fig. 5b-c), which is supported by fossil records from the Pliocene–Pleistocene period in Asia (data from PBDB: https://paleobiodb.org/#/). The pattern of rapid radiation during this period (especially the Pleistocene) has also been observed in many plant groups in East Asia [85, 86], likely due to the effects of glaciation and climatic oscillations during the Pleistocene. East Asia, being less influenced by Quaternary glaciations, had a lower extinction rate or higher speciation rate during those times, leading to a relatively high net diversification rate [87–90]. Biotic factors, such as hybridization/introgression, possibly related to climate oscillations, have also contributed to the emergence of young species during the Pleistocene period. In theory, climate oscillations during the period may have favored introgressive hybridization/introgression upon secondary contact following initial species divergence [91, 92]. Ancestral polymorphism of plastomes could have been purified through the extent of chloroplast-capture events when hybridization/introgression frequently occurred [93], obscuring species boundaries and creating the illusion of young species pairs. Further studies, including nuclear sequences and more samplings, are needed to investigate this hypothesis.

## Conclusions

In this study, we sequenced, assembled, and analyzed the plastomes of 34 Asiatic *Spiraea* accessions. The *Spiraea* plastid genome exhibits typical quadripartite structures and encodes 113–114 genes, including 78–79 protein-coding genes (PCGs), 30 tRNA genes, and 4 rRNA genes. There is a significant correlation between genome size and the length of the SC region. Several hypervariable regions within the *Spiraea* plastome were identified, all of which were localized in the SC regions. Our phylogenomic analysis successfully provides a phylogenetic framework for *Spiraea* but does not support its currently defined section boundaries. Additionally, we discovered that the genus underwent diversification after the Early Oligocene (~ 30 Ma), followed by a rapid speciation process during the Pliocene and Pleistocene periods. Although not perfect, the plastomes of *Spiraea* provide invaluable insights into its phylogenetic relationships and evolutionary history. Further investigations utilizing other genomes, such as the nuclear genome, are urgently needed to enhance our understanding of the evolutionary history of the genus.

### Supplementary Information

**Additional file 1: Fig. S1.** Sequence identity plots of plastomes of *Spiraea* by mVISTA. The top line shows the orientation of genes. A cutoff of 70% identity was used for the plots, and the Y-scale represents the percentage identity ranging from 50 to 100%. **Fig. S2.** Comparison of LSC, IRs, and SSC junction positions among *Spiraea* plastomes. **Fig. S3.** Cladogram of the maximum likelihood (ML) phylogenetic tree constructed based on the whole plastid sequences. Node labels represent the bootstrap values (left) and Bayesian posterior probabilities (right), respectively. **Fig. S4. **Maximum clade-credibility tree of Rosaceae from BEAST with 12 fossil calibration points. The bar at each node indicates age with a 95% height posterior distribution. Red stars represent fossil calibration points (see Table S2). **Table S1.** The sampling information in this study. **Table S2.** Fossil calibration points used in this study. **Table S3. **Plastid genome information of *Spiraea.* **Table S4.** List of annotated genes in the plastid genome of *Spiraea*. **Table S5.** The value of nonsynonymous (*d*_N_), synonymous (*d*_S_) substitution rate and *d*_N_/*d*_S_ in each functional gene. **Table S6.** The likelihood ratio and positively selected codon site tests in this study.

## Data Availability

All newly derived plastid sequences from this study have been deposited in the National Center for Biotechnology Information (NCBI) (see Table S1).
