# Supplementary figures and images for: Plastid genome and its phylogenetic implications of Asiatic Spiraea (Rosaceae)
Source: BMC Plant Biol. 2024 Jan 3;24:23. doi: 10.1186/s12870-023-04697-8 (PMC10763413; doi:10.1186/s12870-023-04697-8)

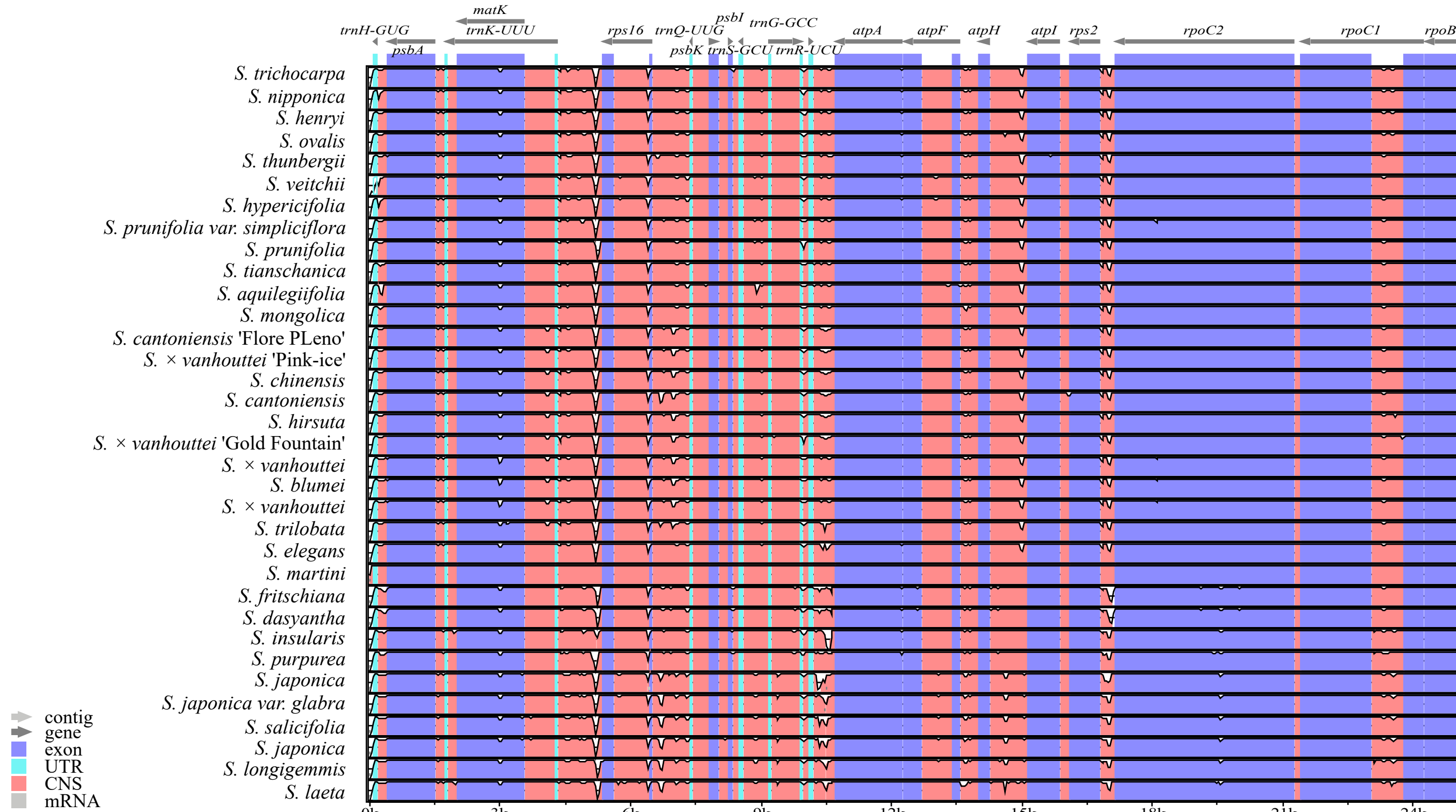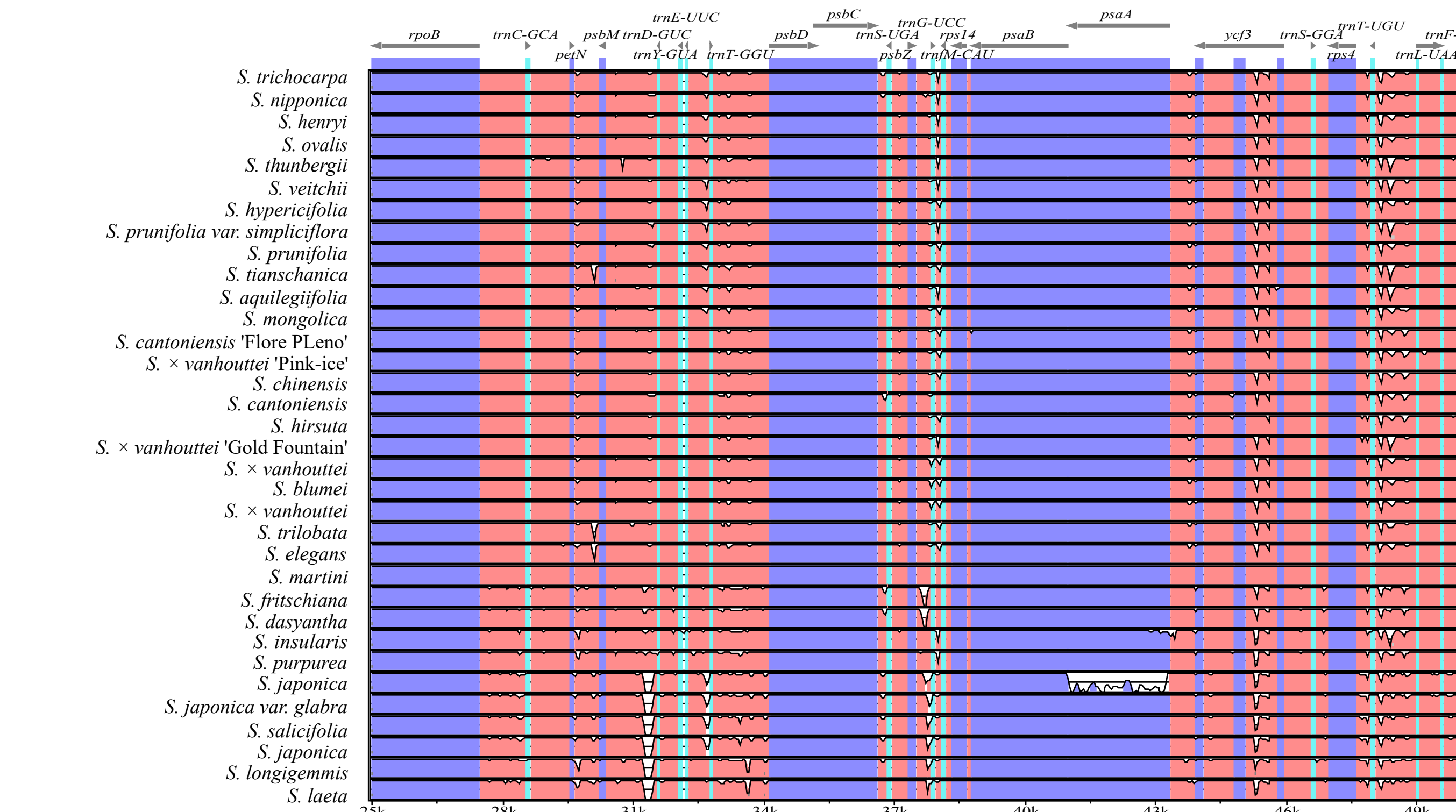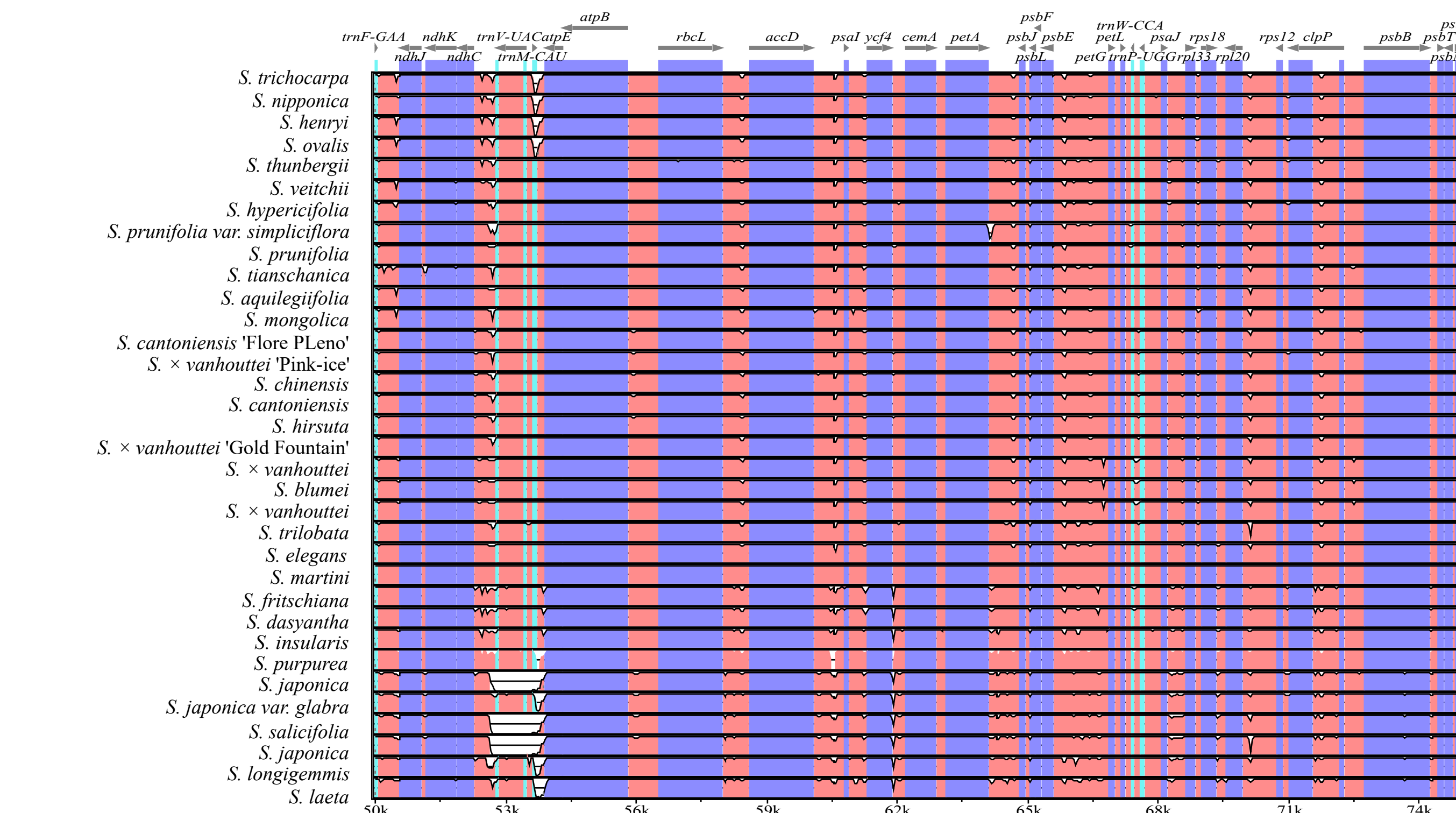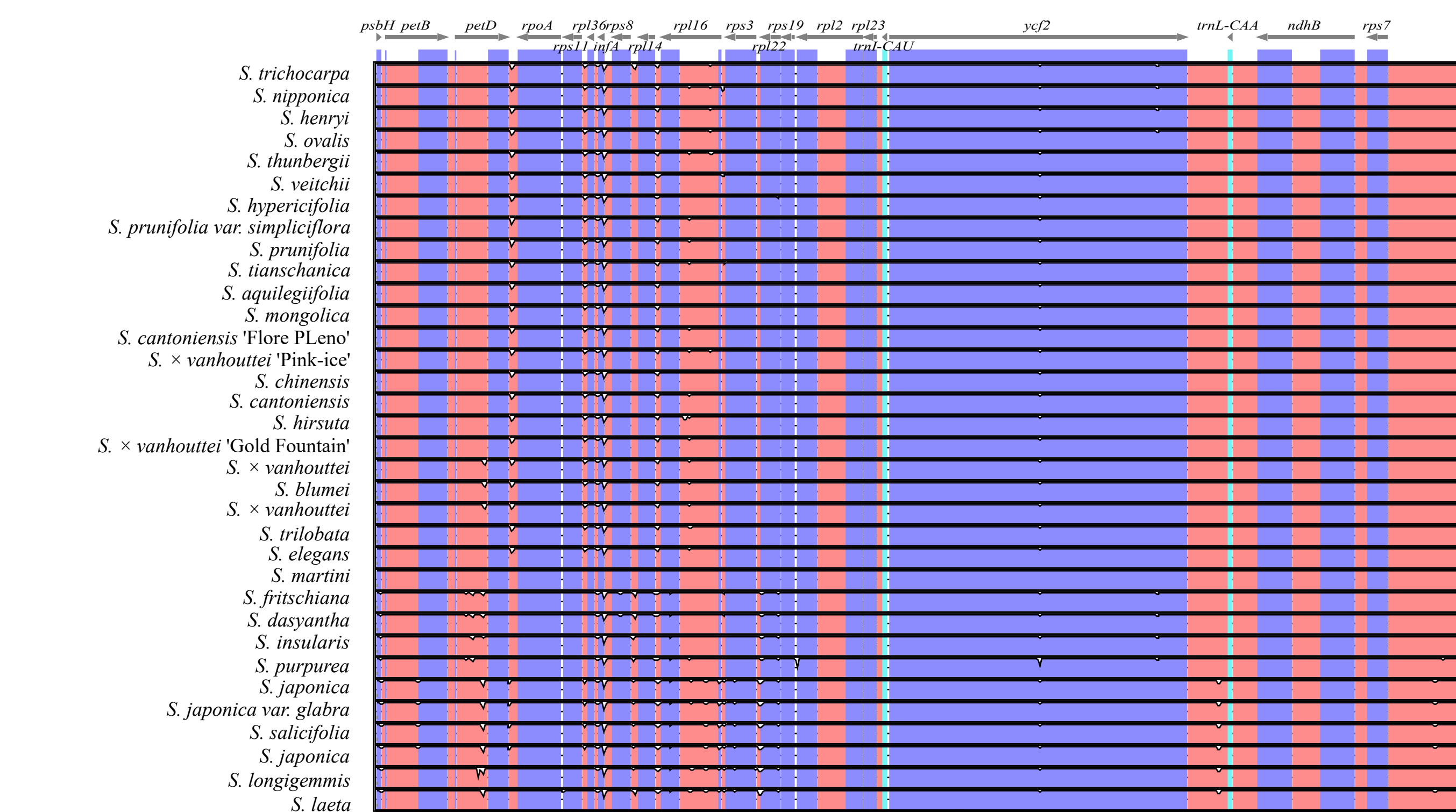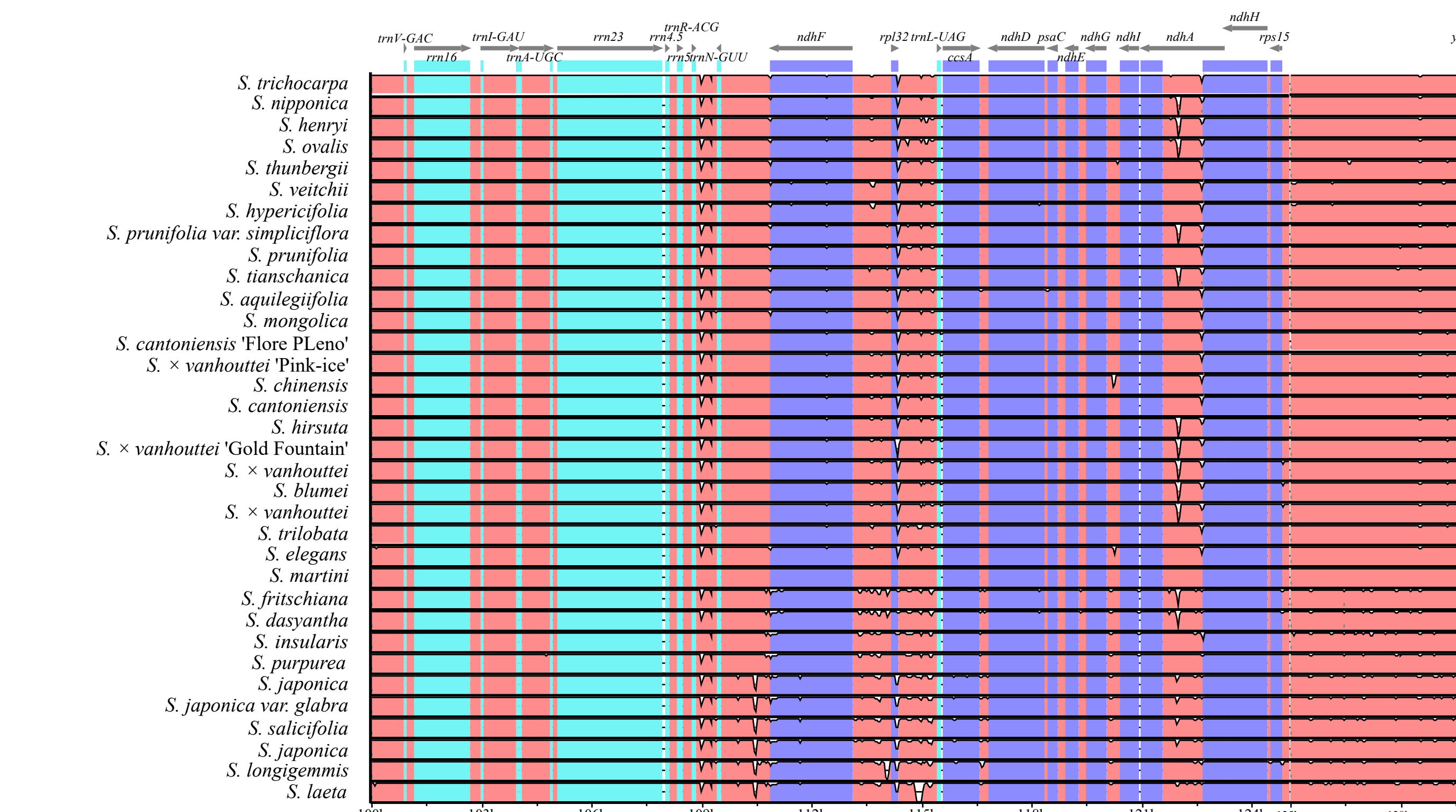

Supplement: Supplementary file 1 — Additional file 1: Fig. S1. Sequence identity plots of plastomes of Spiraea by mVISTA. The top line shows the orientation of genes. A cutoff of 70% identity was used for the plots, and the Y-scale represents the percentage identity ranging from 50 to 100%. Fig. S2. Comparison of LSC, IRs, and SSC junction positions among Spiraea plastomes. Fig. S3. Cladogram of the maximum likelihood (ML) phylogenetic tree constructed based on the whole plastid sequences. Node labels represent the bootstrap values (left) and Bayesian posterior probabilities (right), respectively. Fig. S4. Maximum clade-credibility tree of Rosaceae from BEAST with 12 fossil calibration points. The bar at each node indicates age with a 95% height posterior distribution. Red stars represent fossil calibration points (see Table S2). Table S1. The sampling information in this study. Table S2. Fossil calibration points used in this study. Table S3. Plastid genome information of Spiraea. Table S4. List of annotated genes in the plastid genome of Spiraea. Table S5. The value of nonsynonymous (dN), synonymous (dS) substitution rate and dN/dS in each functional gene. Table S6. The likelihood ratio and positively selected codon site tests in this study. [file 12870_2023_4697_MOESM1_ESM.zip › Fig. S1.pdf]

Clade I

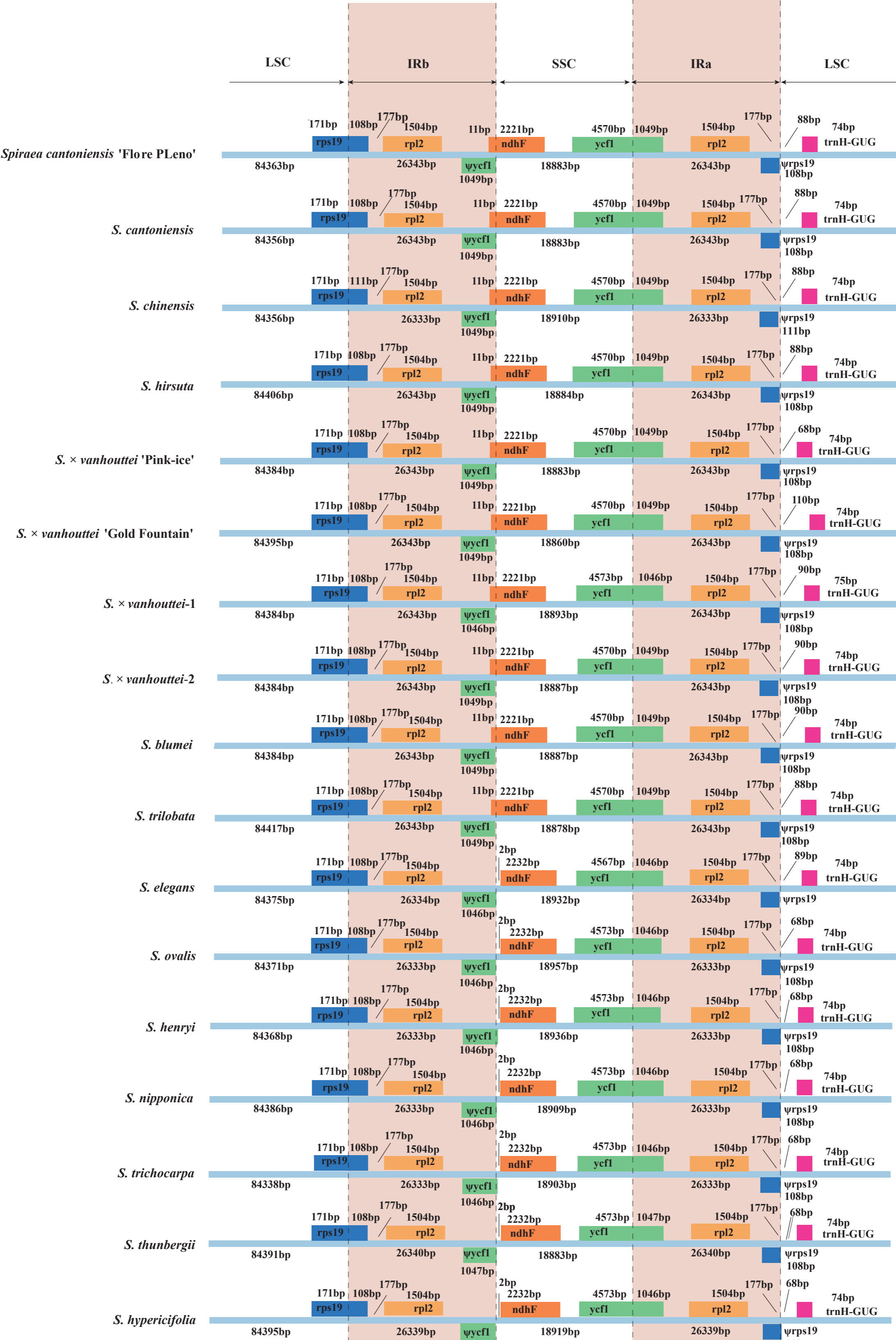

Clade II

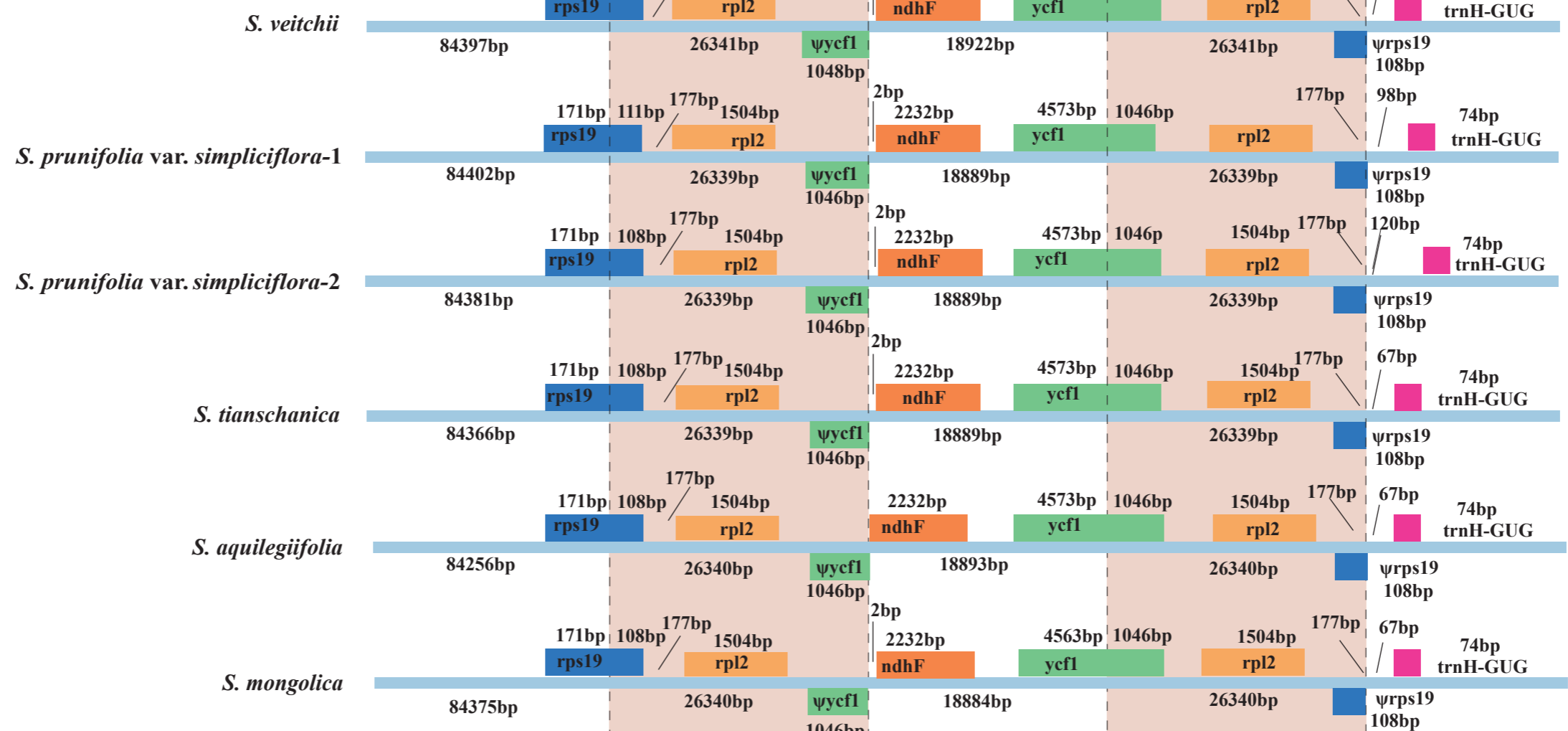

Clade III

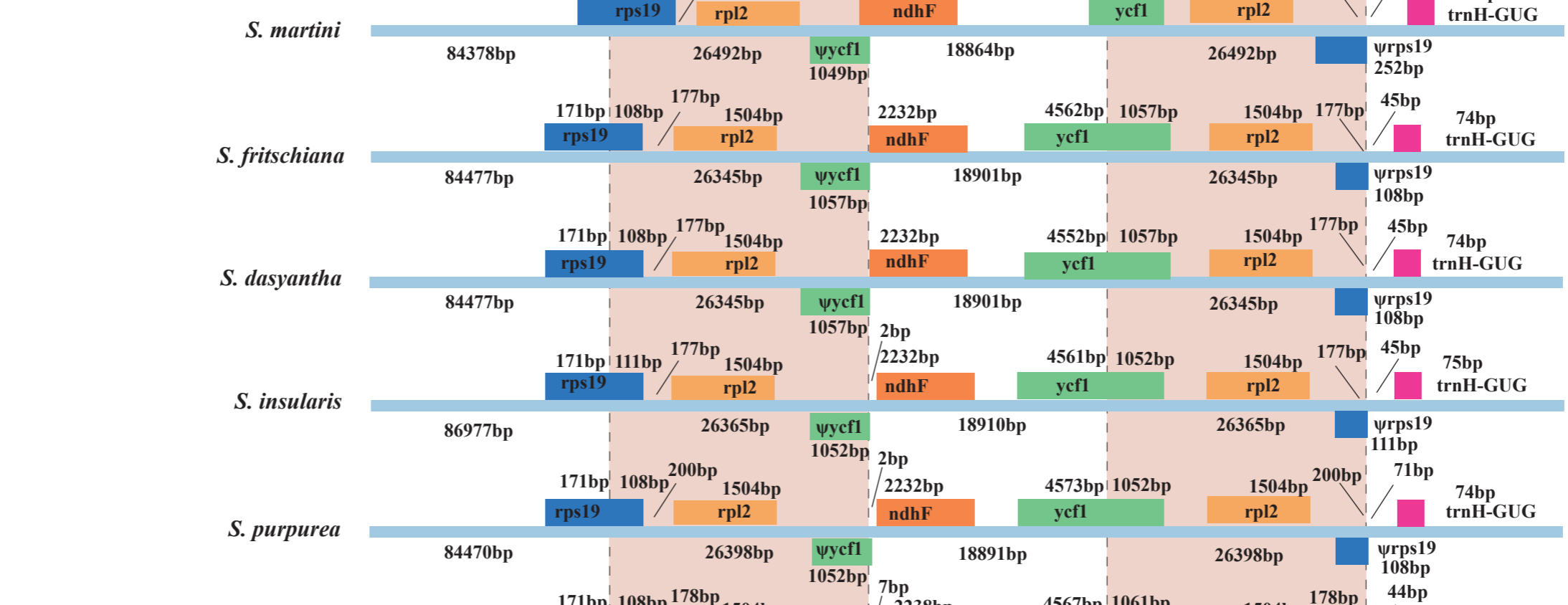

Clade IV

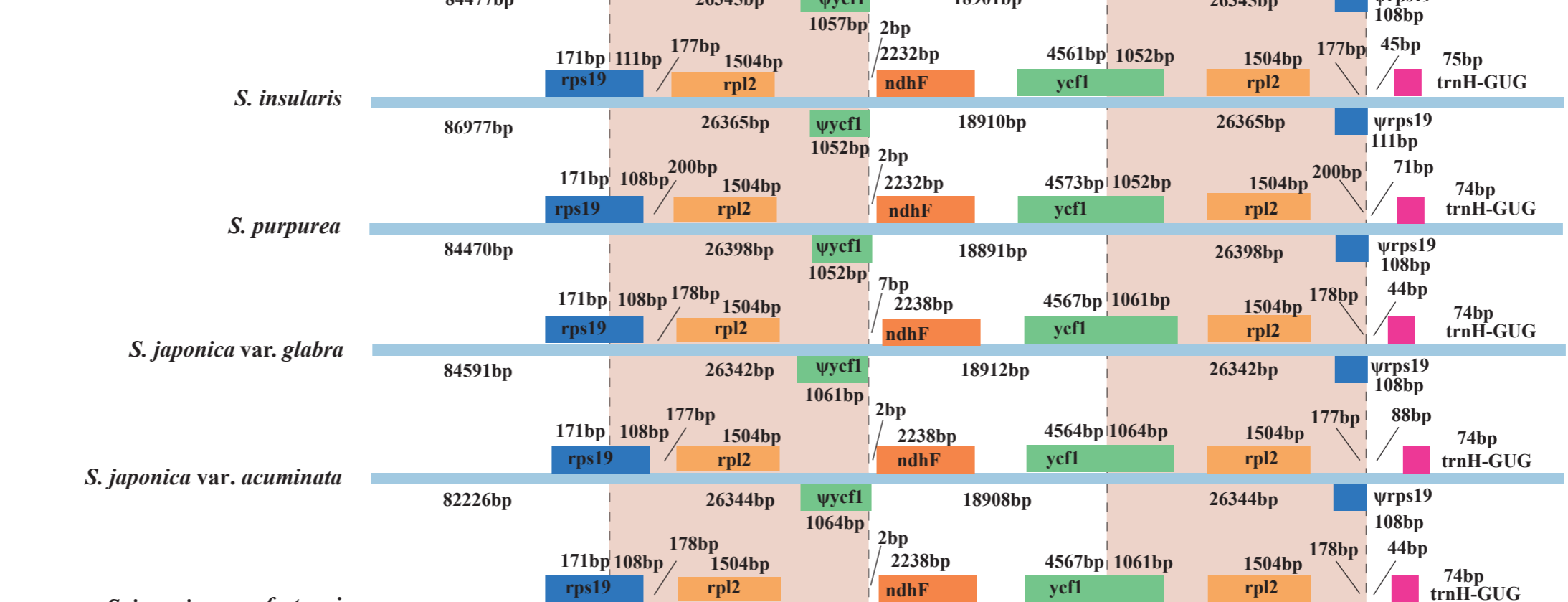

Clade V

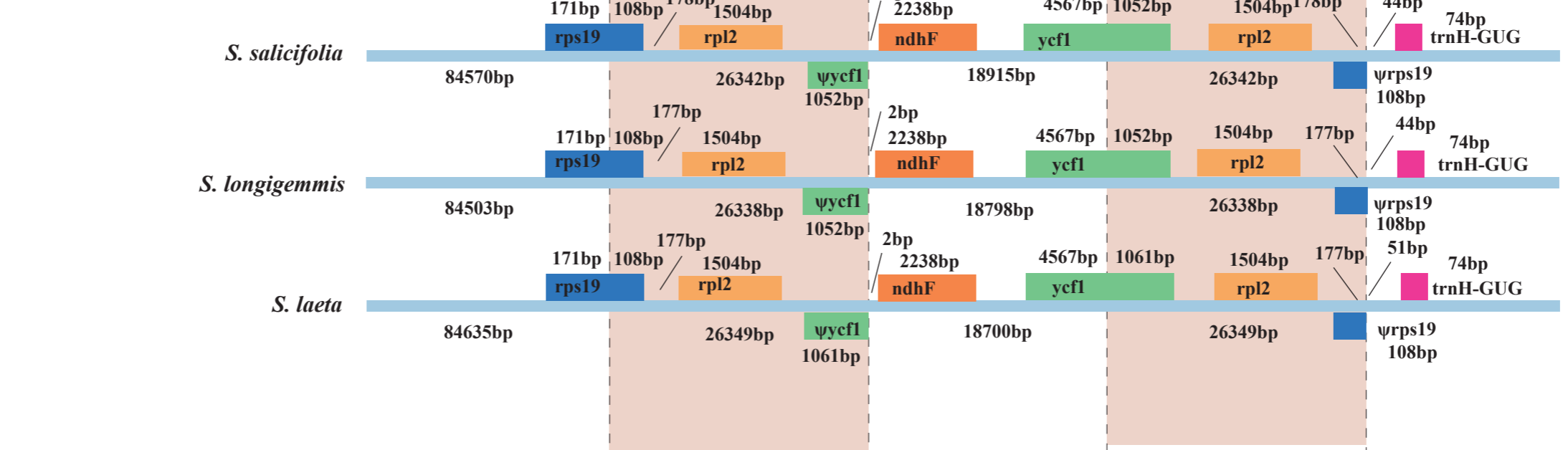

Supplement: Supplementary file 1 — Additional file 1: Fig. S1. Sequence identity plots of plastomes of Spiraea by mVISTA. The top line shows the orientation of genes. A cutoff of 70% identity was used for the plots, and the Y-scale represents the percentage identity ranging from 50 to 100%. Fig. S2. Comparison of LSC, IRs, and SSC junction positions among Spiraea plastomes. Fig. S3. Cladogram of the maximum likelihood (ML) phylogenetic tree constructed based on the whole plastid sequences. Node labels represent the bootstrap values (left) and Bayesian posterior probabilities (right), respectively. Fig. S4. Maximum clade-credibility tree of Rosaceae from BEAST with 12 fossil calibration points. The bar at each node indicates age with a 95% height posterior distribution. Red stars represent fossil calibration points (see Table S2). Table S1. The sampling information in this study. Table S2. Fossil calibration points used in this study. Table S3. Plastid genome information of Spiraea. Table S4. List of annotated genes in the plastid genome of Spiraea. Table S5. The value of nonsynonymous (dN), synonymous (dS) substitution rate and dN/dS in each functional gene. Table S6. The likelihood ratio and positively selected codon site tests in this study. [file 12870_2023_4697_MOESM1_ESM.zip › Fig. S2.pdf]

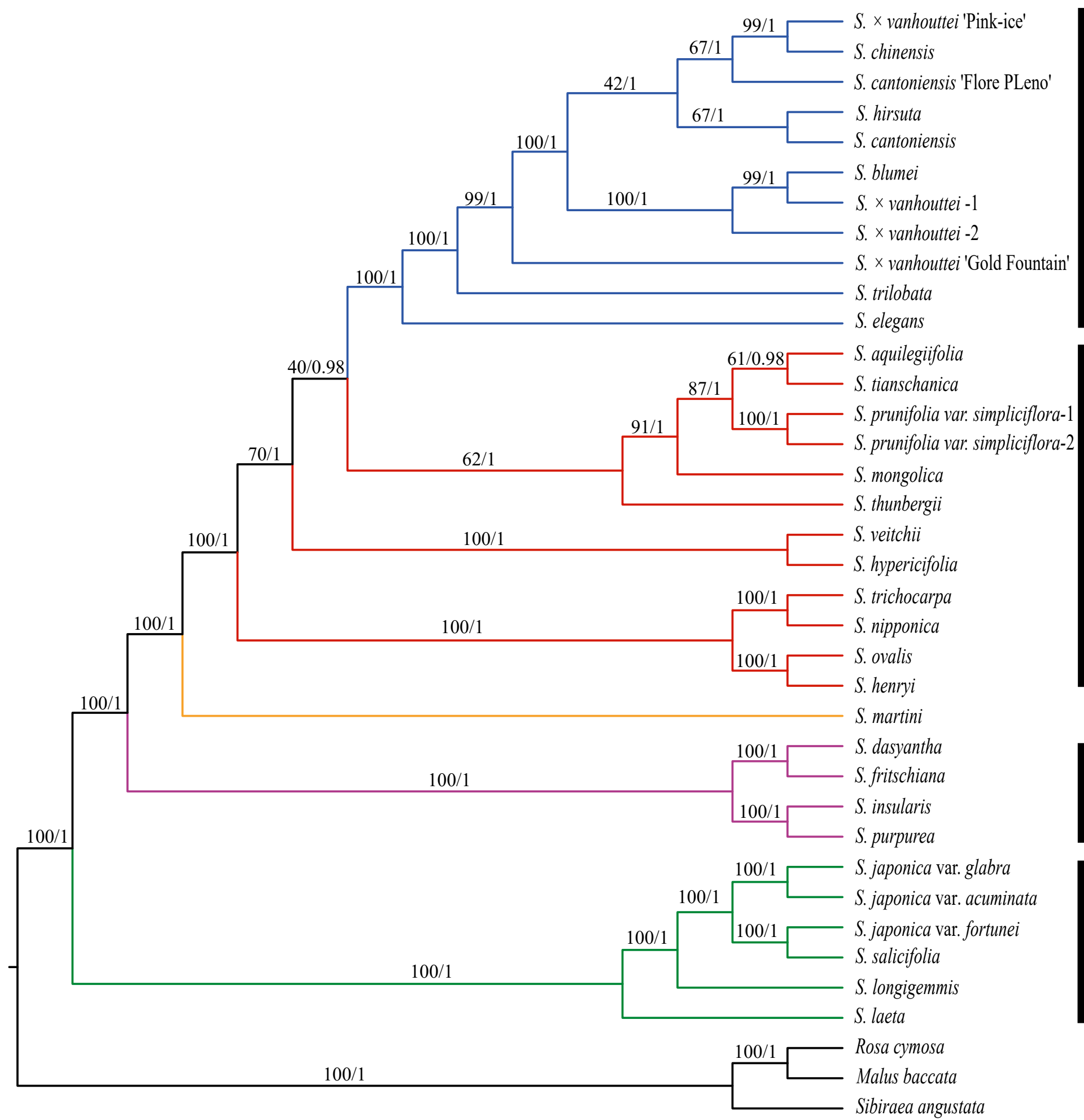

Clade I

Clade II

Clade III

Clade IV

Clade V

Supplement: Supplementary file 1 — Additional file 1: Fig. S1. Sequence identity plots of plastomes of Spiraea by mVISTA. The top line shows the orientation of genes. A cutoff of 70% identity was used for the plots, and the Y-scale represents the percentage identity ranging from 50 to 100%. Fig. S2. Comparison of LSC, IRs, and SSC junction positions among Spiraea plastomes. Fig. S3. Cladogram of the maximum likelihood (ML) phylogenetic tree constructed based on the whole plastid sequences. Node labels represent the bootstrap values (left) and Bayesian posterior probabilities (right), respectively. Fig. S4. Maximum clade-credibility tree of Rosaceae from BEAST with 12 fossil calibration points. The bar at each node indicates age with a 95% height posterior distribution. Red stars represent fossil calibration points (see Table S2). Table S1. The sampling information in this study. Table S2. Fossil calibration points used in this study. Table S3. Plastid genome information of Spiraea. Table S4. List of annotated genes in the plastid genome of Spiraea. Table S5. The value of nonsynonymous (dN), synonymous (dS) substitution rate and dN/dS in each functional gene. Table S6. The likelihood ratio and positively selected codon site tests in this study. [file 12870_2023_4697_MOESM1_ESM.zip › Fig. S3.pdf]

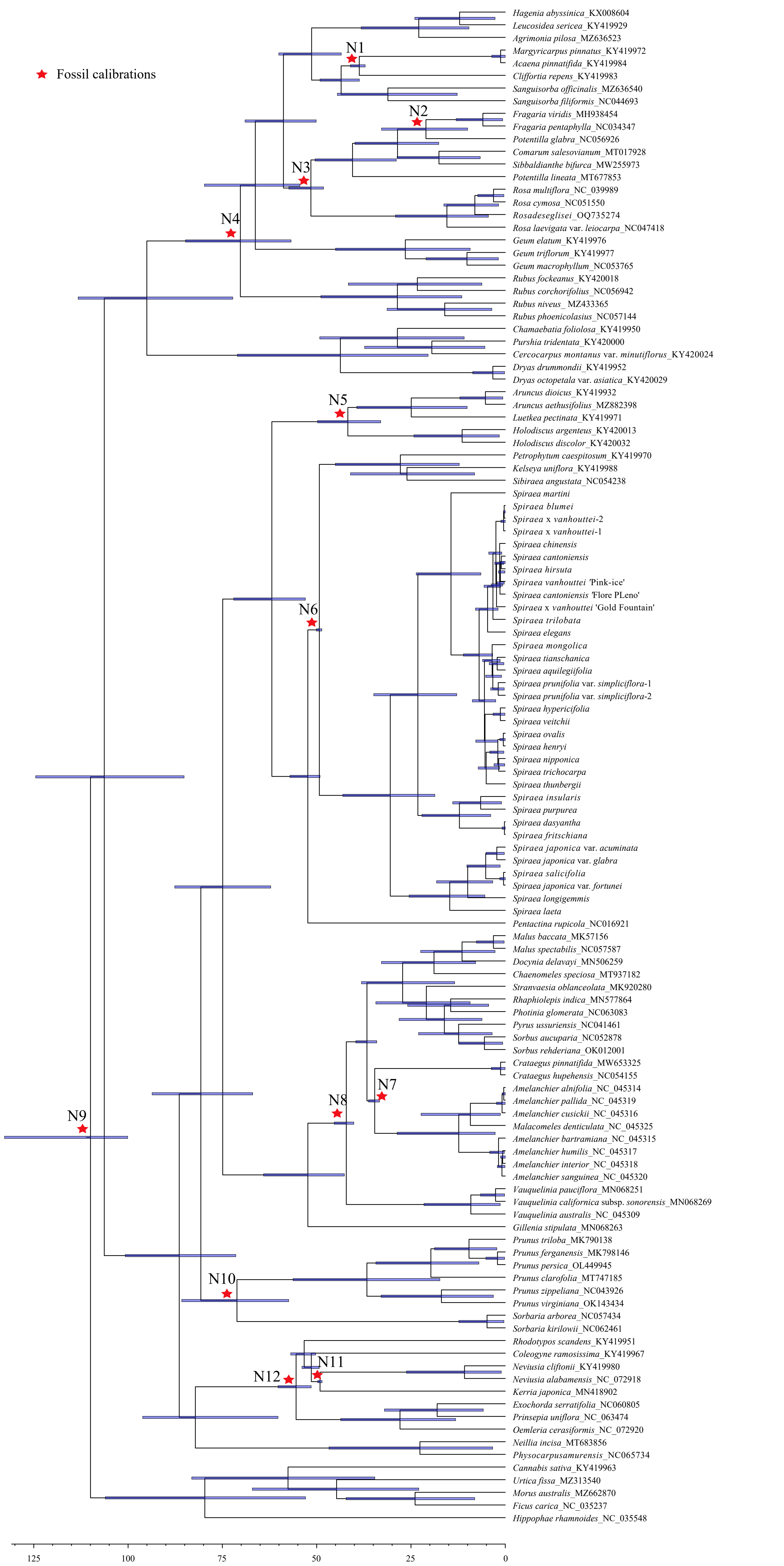

Supplement: Supplementary file 1 — Additional file 1: Fig. S1. Sequence identity plots of plastomes of Spiraea by mVISTA. The top line shows the orientation of genes. A cutoff of 70% identity was used for the plots, and the Y-scale represents the percentage identity ranging from 50 to 100%. Fig. S2. Comparison of LSC, IRs, and SSC junction positions among Spiraea plastomes. Fig. S3. Cladogram of the maximum likelihood (ML) phylogenetic tree constructed based on the whole plastid sequences. Node labels represent the bootstrap values (left) and Bayesian posterior probabilities (right), respectively. Fig. S4. Maximum clade-credibility tree of Rosaceae from BEAST with 12 fossil calibration points. The bar at each node indicates age with a 95% height posterior distribution. Red stars represent fossil calibration points (see Table S2). Table S1. The sampling information in this study. Table S2. Fossil calibration points used in this study. Table S3. Plastid genome information of Spiraea. Table S4. List of annotated genes in the plastid genome of Spiraea. Table S5. The value of nonsynonymous (dN), synonymous (dS) substitution rate and dN/dS in each functional gene. Table S6. The likelihood ratio and positively selected codon site tests in this study. [file 12870_2023_4697_MOESM1_ESM.zip › Fig. S4.pdf]
